# The Impact of Immune-Modulating Treatments for Dermatological Diseases on the Risk of Infection with SARS-CoV-2 and Outcomes Associated with COVID-19 Illness

**DOI:** 10.1007/s13671-023-00385-w

**Published:** 2023-04-03

**Authors:** Nicole Maynard, April W. Armstrong

**Affiliations:** Keck School of Medicine of USC, 1975 Zonal Avenue, KAM 510, MC 9034, Los Angeles, CA 90089 USA

**Keywords:** COVID-19, Biologics, Immune-modulating treatments, Immune-mediated skin conditions

## Abstract

***Purpose of Review*:**

Immune-modulating treatments are used in dermatology for a variety of conditions. The authors aim to review the data regarding the safety of these treatments during the COVID-19 pandemic, namely the risk of infection with SARS-CoV-2 and the outcomes associated with COVID-19-related illness.

***Recent Findings*:**

Several large-scale studies found no increased risk of COVID-19 infection for patients on TNF-α inhibitors, IL-17 inhibitors, IL-12/23 inhibitors, IL-23 inhibitors, dupilumab, and methotrexate. They also found that these patients did not have worse outcomes when infected with COVID-19. The data regarding JAK inhibitors, rituximab, prednisone, cyclosporine, mycophenolate mofetil, and azathioprine are more mixed.

***Summary*:**

Based on current research and guidelines from the American Academy of Dermatology and the National Psoriasis Foundation, dermatology patients on immune-modulating therapies can continue treatment during the COVID-19 pandemic when they are not infected with SARS-CoV-2. For patients who have COVID-19, guidelines encourage individualized assessment of the benefits and risks of continuing or temporarily withholding treatment.

## Introduction

Systemic therapies are a valuable treatment option for a number of different immune-mediated dermatological diseases. For the past 2 decades, immune-modulating medications have become particularly important as treatment options for chronic inflammatory skin diseases, such as psoriasis, atopic dermatitis, hidradenitis suppurativa, bullous pemphigoid, pemphigus, and many other conditions.

Most immunomodulators used in dermatology target specific steps in the immune pathogenesis of the skin disease. These targets include cytokines such as tumor necrosis factor (TNF), IL-4, IL-12, IL-13, IL-17, IL-23, and their receptors, as well as intracellular molecules such as those involved in the JAK-STAT pathway [1, 2].

With the COVID-19 pandemic, the safety profiles of these immunomodulatory agents will need to be closely examined to understand their interaction with COVID-19 infection. This article will review the current data on the impact of immune-modulating treatments used for dermatological conditions on the risk of contracting SARS-CoV-2 and the severity of COVID-19-related outcomes.

## Biologics

Compared to other therapies in dermatology, biologics used for immune-mediated dermatological diseases have been more extensively studied during the COVID-19 pandemic. The quality of evidence varies among different studies [3•, 4, 5•, 6–8]. A few studies focusing on biologics in patients with psoriasis, and their findings are mixed. For example, in a 2020 retrospective observational study in Verona, Italy, researchers investigated the risk of COVID-19 hospitalization or death in psoriasis patients on biologics. Compared to the general population, psoriasis patients on biologics did not have increased risks of hospitalization or death [3•]. However, a single-center case-control study in the Lombardia region of Italy found that psoriatic patients on biologics had a higher risk of testing positive for COVID-19 compared to non-psoriatic individuals in this region. They also showed increased risk of hospitalization but notably did not have in increased risk of ICU admission or death [9•]. Additional research has focused on assessing COVID-19 risks and outcomes within specific biologic cohorts, which are discussed as follows.

### TNF- α Inhibitors

Tumor necrosis factor α inhibitors (TNFi) are a class of biologics approved to treat immune-mediated skin diseases, such as moderate-to-severe plaque psoriasis and hidradenitis suppurativa [10]. FDA-approved medications in this class for dermatological indications include adalimumab, certolizumab, etanercept, and infliximab. These TNF inhibitors have direct inhibitory effects on TNF-α, which is involved in the inflammatory pathways that contribute to the etiology of psoriasis, hidradenitis suppurativa, and a number of other inflammatory dermatological conditions.

Before the data for COVID-19 became available, the research community first focused on the relationship between TNFi and the risk of respiratory tract infections. A meta-analysis of phase 3 clinical trial data assessed the impact of TNFi on the risk of respiratory tract infections (RTI) [11]. Overall, researchers did not find a significantly increased risk of RTI in patients on TNFi therapy compared to placebo (Odds ratio [OR], 1.08; 95% CI, 0.84–1.38; *p* = 0.55) [11]. In terms of COVID-19 risk, a retrospective matched cohort study in Massachusetts found that patients on TNFi therapy for the rheumatic and inflammatory disease actually had a lower likelihood of being diagnosed with COVID-19 compared to matched controls (OR 0.69, 95% CI 0.48–0.98,82 *p* = 0.04) [4].

TNF-α inhibitors do not appear to affect the severity of COVID-19 infection in patients with immune-mediated diseases [5, 12]. In certain populations, the use of TNFi may even suggest a beneficial impact. In a meta-analysis of 35 studies, patients with the rheumatic or inflammatory disease on TNF inhibitors had a lower probability of severe COVID-19 disease (pooled OR = 0.63, 95% CI: 0.41–0.96, *I*2 = 0) and a nearly 50% lower probability of hospitalization (pooled OR = 0.53, 95% CI: 0.42–0.67, *I*2 = 0) when compared to patients not taking TNF inhibitors (adjusted for age, sex, and comorbidities) [5•]. In a cohort study of patients with immune-mediated diseases, including psoriasis, patients on TNF inhibitor monotherapy had lower risk of COVID-19-related hospitalization or death compared to individuals on combination regimens or monotherapy with methotrexate, azathioprine/6-mercaptopurine, and Jak inhibitors [12]. A U.S. retrospective cohort study also found that rheumatoid arthritis (RA) patients with COVID-19 on TNFi had a lower risk of hospitalization compared to RA patients on non-TNFi biologics (OR 0.32, 95% CI 0.20–0.53) and the general population (OR 0.77, 95% CI 0.51–1.17) [13]. These results suggest potential protective effects of TNFi therapy.

These findings are consistent with research suggesting TNF-α plays a significant role in the pathogenesis of severe disease in COVID-19 [14]. In fact, one study showed improvement in severe COVID-19 with TNFi treatment. In a clinical trial, 18 patients with severe COVID-19 were treated with infliximab-abda, a TNF inhibitor. Of the subjects, 16 (89%) achieved sustained improvement in SpO2/FiO2 of ≥ 50 for at least 48 h, the primary endpoint of the trial [15]. Further research needs to be done on the exact role of TNF-ɑ antagonism in COVID-19 treatment.

Based on the aforementioned literature, several organizations have released guidelines for the treatment of various skin conditions during the COVID-19 pandemic [16, 17••, 18••, 19]. These guidelines are summarized in Table 1.

**Table 1:** Dermatologic society and organization recommendations regarding the use of immunomodulating therapies during the COVID-19 pandemic

	**Recommendations for patients who do not have active SARS-CoV-2 infection**	**Recommendations for patients with suspected/confirmed COVID-19**
TNF-alpha inhibitors, IL-17 inhibitors, IL-23 inhibitors, IL-12/23 inhibitors	**National Psoriasis Foundation (NPF):** continue systemic therapy **Hidradenitis Suppurativa Foundation (HSF):** continue systemic therapy **American Academy of Dermatology (AAD):** continue systemic therapy	**NPF:** discuss with a healthcare provider to discuss the risks and benefits of continuing biologic therapy **HSF:** discontinue or postpone treatment until symptoms and infection have resolved **AAD:** discontinue or postpone treatment until symptoms and infection have resolved
Dupilumab	**International Eczema Council (IEC):** continue treatment **AAD:** continue treatment	**IEC:** continue therapy for patients who are mildly ill or asymptomatic with a COVID-19 infection. For those who are more symptomatic, reduce the dose or discontinue treatment **AAD:** discontinue systemic therapy and then re-initiate once infection is cleared
JAK inhibitors	**NPF:** continue systemic therapy **AAD:** continue systemic therapy	**NPF:** discuss with a healthcare provider to discuss the risks and benefits of continuing biologic therapy **AAD:** discontinue or postpone treatment until symptoms and infection have resolved
Rituximab	**AAD:** continue systemic therapy	**AAD:** discontinue or postpone treatment until symptoms and infection have resolved
Prednisone	**NPF:** chronic corticosteroid use should be avoided; if they must be continued, the dose should be tapered to the lowest dose necessary to achieve the desired therapeutic effect **IEC:** continue treatment **AAD:** continue systemic therapy	**NPF:** chronic corticosteroid use should be avoided; if they must be continued, the dose should be tapered to the lowest dose necessary to achieve the desired therapeutic effect **IEC:** continue therapy for patients who are mildly ill or asymptomatic with a COVID-19 infection. For those who are more symptomatic, reduce the dose or discontinue treatment **AAD:** discontinue or postpone treatment until symptoms and infection have resolved
Methotrexate	**NPF:** continue systemic therapy **AAD:** continue systemic therapy	**NPF:** discuss with a healthcare provider to discuss the risks and benefits of continuing biologic therapy **AAD:** discontinue or postpone treatment until symptoms and infection have resolved
Cyclosporine	**NPF:** continue systemic therapy **AAD:** continue systemic therapy	**NPF:** discuss with a healthcare provider to discuss the risks and benefits of continuing biologic therapy **AAD:** discontinue or postpone treatment until symptoms and infection have resolved
Mycophenolate mofetil	**NPF:** continue systemic therapy **AAD:** continue systemic therapy	**NPF:** discuss with a healthcare provider to discuss the risks and benefits of continuing biologic therapy **AAD:** discontinue or postpone treatment until symptoms and infection have resolved
Azathioprine	**NPF:** continue systemic therapy **AAD:** continue systemic therapy	**NPF:** discuss with a healthcare provider to discuss the risks and benefits of continuing biologic therapy **AAD:** discontinue or postpone treatment until symptoms and infection have resolved

### IL-17 Inhibitors

IL-17 inhibitors (IL-17i) are in a well-established class of biologics that are highly effective in treating psoriasis [20]. This class includes three FDA-approved medications for psoriasis: secukinumab, brodalumab, and ixekizumab. Some researchers theorize that IL-17 may contribute to COVID-19 pathogenesis and inflammation in the lungs [6], and as such, it is important to investigate the impact of taking IL-17 inhibitors on the risk of infection and severity of COVID-19 disease.

Pre-pandemic studies investigated the risk of respiratory tract infections in patients on IL-17 inhibitors. A meta-analysis of phase 3 clinical trials investigating IL-17i for psoriasis found an increased risk of RTI in patients taking secukinumab, brodalumab, or ixekizumab (odds ratio [OR], 1.56; 95% confidence interval, 1.04–2.33) [21]. However, this study cited shortcomings in their study design due to lack of consistent subjective reporting and unreported etiology of each RTI. Additionally, a response paper by Blauvelt et al. argue that these findings are skewed by both reporting bias and by the incidence of mucocutaneous candidiasis, which may be improperly reported by patients as an RTI [22].

In contrast, a large-scale cohort study in Israel found that the use of IL-17 inhibitors in patients with psoriasis was not associated with increased risk of COVID-19 infection (adjusted HR for IL-17i vs. methotrexate: 0.91 [95% CI, 0.48–1.72]; IL-17I vs. non-systemic/non-immunomodulatory treatments: 0.92 [95% CI, 0.54–1.59]) [23]. The same cohort study also investigated the risk of COVID-related hospitalization and mortality in patients on IL-17 inhibitors. Risk of hospitalization and death due to COVID-19 was comparable between patients on IL-17i when compared to both methotrexate [hospitalization: adjusted HR: 0.42 (95% CI, 0.05–3.39); mortality: 7.57 (95% CI, 0.36–157.36)] and non-systemic/non-immunomodulatory treatments [hospitalization: adjusted HR 0.65 (95% CI, 0.09–4.59); mortality: 7.05 (95% CI, 0.96–51.98)] [23•]. These findings are similar to a New York case series that found no increased risk of hospitalization due to COVID-19 in individuals with the immune-mediated inflammatory disease on IL-17 inhibitors compared to the general population (OR 0.97; 95% CI, 0.72–1.31) [24•].

In general, IL-17 inhibitor therapy does not seem to increase the risk of SARS-CoV-2 infection or worsen outcomes compared to those not on IL-17i. Organizational guidelines on the use of systemic medications during COVID-19 are summarized in Table 1.

### IL-12/23 Inhibitors

At this time, ustekinumab is the only FDA-approved IL-12/23 inhibitor for psoriasis. It targets the shared p40 protein subunit to limit the downstream activation of inflammatory mediators in the pathogenesis of psoriasis [25].

Previous studies have investigated the risk of RTI in patients on IL-12/23 inhibitors. A meta-analysis of phase 3 clinical trials found an increased risk of upper respiratory infections (Mantel-Haenszel Risk Difference [MH RD] 0.028; 95% CI, 0.004–0.053; *p* = .024) in participants with inflammatory and rheumatic diseases or healthy volunteers. However, treatment with these medications did not increase the risk of specifically viral upper RTI or lower RTI [26]. It is notable that one study found that patients on a TNFi had a decreased risk of COVID-19 infection compared to patients on ustekinumab (fully-adjusted HR, 0.04; 95% CI, 0.00–0.64; *p* = 0.022) [27]. Thus, while IL-12/23 inhibitors may not increase the risk of RTIs, they are also not protective against COVID-19.

Limited case reports have been published regarding patients on ustekinumab who contract SARS-CoV-2 [28]. A case series from New York University looked at 86 patients with the immune-mediated inflammatory disease who contracted Sars-CoV-2, 6 of whom were on an IL-12/23 inhibitor. There was no increase in COVID-19-related hospitalization within this group (OR 0.86; 95% CI, 0.65–1.15) [24•]. In the previously mentioned study comparing ustekinumab to TNFi, TNFi patients had a reduced risk of hospitalization compared to patients on ustekinumab [27]. Again, this reaffirms that unlike ustekinumab does not provide the same protective effects as TNFi.

Based on the aforementioned evidence, IL-12/23 inhibitors do not seem to increase the risk of SARS-CoV-2 infection or COVID-19-related hospitalization. However, it does not provide a protective effect either. Organizational guidelines on the use of systemic medications during COVID-19, as well as vaccination recommendations, are summarized in Table 1.

### IL-23 Inhibitors

IL-23 inhibitors target the p19 subunit, reducing Th17 pathway activity [25]. The approved IL-23 inhibitors for psoriasis are guselkumab, tildrakizumab, and risankizumab.

As previously discussed, a meta-analysis of phase 3 clinical trials assessed whether patients on IL-12/23 or IL-23 inhibitors experienced an increased risk of respiratory infections. They found an increased risk of upper respiratory infections (Mantel-Haenszel Risk Difference [MH RD] 0.028; 95% CI, 0.004–0.053; *p* = .024). However, treatment with these medications did not increase the risk of viral upper RTI or lower RTI [26]. In another study, which analyzed phase 3 clinical trials for subjects with psoriasis on IL-23 inhibitors, they did not find a statistically significant increase in the risk of RTI (Odds ratio, 1.24; 95% confidence interval, 0.98–1.56; *p* = .07) [29].

Limited data exists on the impact of IL-23 inhibitors and their impact on COVID-19 outcomes. The same New York–based case series discussed previously also included patients with the immune-mediated inflammatory disease on IL-23 inhibitors. There was no increase in the risk of hospitalization due to COVID-19 in this cohort (OR 0.75; 95% CI, 0.50–1.13) [24•].

These findings suggest that it is likely safe to continue taking IL-23 inhibitors during the COVID-19 pandemic. Organizational guidelines on the use of systemic medications during COVID-19, as well as vaccination recommendations, are summarized in Table 1.

### Dupilumab

Dupilumab is FDA-approved for the treatment of atopic dermatitis (AD). The pathogenesis of AD involves a predominance in Th2 immunity, leading to elevated IL-4 levels. Increased IL-4 then inhibits Th1 [30]. There is evidence that this disruption of the Th1/Th2 ratio leads to increased susceptibility to viral infections [8]. This has been demonstrated in COVID-19 infections as well: an increased Th2/Th1 ratio is associated with a poorer prognosis [31]. Dupilumab, a first-line treatment for AD, modulates the Th1/Th2 axis disruption. It works by targeting the IL-4 alpha chain, which inhibits both IL-4 and IL-13 signaling pathways [32].

Studies have investigated the risk of infections, namely RTI, in patients on dupilumab. In a meta-analysis of three phase 3 clinical trials, patients with atopic dermatitis on dupilumab did not have increased rates of overall infections, upper RTI, or nasopharyngitis [33]. Studies have also specifically investigated dupilumab and COVID-19. In a population-based cohort study, AD patients on dupilumab were compared to patients on topical corticosteroids, phototherapy, and azathioprine or mycophenolate mofetil in regard to COVID-19 incidence and outcomes. They found that dupilumab was not associated with an increase in SARS-CoV-2 infection risk [adjusted HR for dupilumab vs. prolonged systemic corticosteroids: 1.13 (95% CI, 0.61–2.09); dupilumab vs. phototherapy: 0.80 (95% CI, 0.42–1.53); dupilumab vs. azathioprine/MMF: 1.10 (95% CI, 0.45–2.65)] [34•]. The same cohort study also found no increased risk of hospitalization [adjusted HR for dupilumab vs. prolonged systemic corticosteroids: 0.35 (95% CI, 0.05–2.71); dupilumab vs. phototherapy: 0.43 (95% CI, 0.05–3.98); dupilumab vs. azathioprine/MMF: 0.25 (95% CI, 0.02–2.74)] or, when applicable, mortality [HR for dupilumab vs. prolonged systemic corticosteroids: 0.04 (95% CI, 0.00–225.20)] due to COVID-19 in patients using dupilumab [34•]. In a prospective registry study, AD patients on dupilumab had a lower likelihood of experiencing moderate-to-severe COVID-19 symptoms compared to those using other systemic treatments (odds ratio [OR] = 3.89; *p* = .008) or those using limited to no treatment. (OR = 1.96; *p* = .04) [35]. This is hypothesized to be in part due to regulation of the Th2 response.

Based on these findings, dupilumab can be safely continued during the COVID-19 pandemic. The International Eczema Council (IEC) guidelines regarding its use during the pandemic are included in Table 1.

### JAK Inhibitors

The Janus kinase and signal transducer and activator of transcription (JAK-STAT) pathway is heavily utilized by a variety of inflammatory cytokines and processes [36]. JAK inhibitors target the various JAK proteins and have proven effective in the treatment of a variety of dermatological diseases, including psoriasis, atopic dermatitis, vitiligo, and alopecia areata [35]. However, the only FDA-approved JAK inhibitors for dermatologic indications are upadacitinib and abrocitinib, both of which are approved for atopic dermatitis. Upadacitinib and tofacitinib are approved for psoriatic arthritis, and tofacitinib has also been shown to be effective for plaque psoriasis [37].

Limited data exists regarding the association between SARS-CoV-2 infection risk and JAK inhibitor use. A meta-analysis of phase II and III clinical trials did not find a statistically significant increased risk of serious infection for patients with rheumatoid arthritis on tofacitinib (IRR 1.22 [95% CI: 0.60, 2.45]), baricitinib (IRR 0.80 [95% CI: 0.46, 1.38]), and upadacitinib (IRR 1.14 [95% CI: 0.24, 5.43]) versus placebo [38]. However, upper respiratory infections were the second most common adverse event reported in two replicate double-blind, randomized controlled phase 3 trials investigating upadacitinib in patients with atopic dermatitis (11% vs. 7% placebo; 11.5% vs 4% placebo) [39].

Furthermore, data is conflicting whether JAK inhibitors are beneficial or harmful in COVID-19-associated prognosis. A German registry study of patients with rheumatologic diseases found that JAK inhibitors were associated with more severe COVID-19-related outcomes (OR 1.8, 95% CI 1.1 to 2.7) [40]. However, in a New York-based case series, no increased risk of hospitalization was found for patients on JAK inhibitors compared to the general population (OR 1.05; 95% CI, 0.78–1.41) [24]. Another study that analyzed COVID-19 outcomes across three registries (Global Rheumatology Alliance, PsoProtect, and SECURE-IBD) found in a pooled analysis that, compared to TNFi monotherapy, patients on JAK inhibitors had higher odds of hospitalization or death (OR, 1.82; 95% CI, 1.21–2.73; *p* = .004) [12]. However, in another study of the SECURE-IBD registry that investigated JAK inhibitors compared to other IBD therapies, there was no significant difference in hospitalization (21.6% vs 23.3%), ICU admission (5.4% vs 4.5%), or severe COVID-19 (6.2% in both groups) [41]. The evidence seems to suggest that, while higher risk than TNFi, JAK inhibitors do not necessarily increase the risk of hospitalization or severe COVID-10 compared to the general population.

Despite the mixed evidence, there are no specific guidelines published regarding the use of JAK inhibitors during the COVID-19 pandemic for dermatological conditions. The general guidelines for systemic medications from the AAD and NPF are outlined in Table 1.

### Rituximab

Rituximab is an anti-CD20 antibody that is FDA-approved for dermatologic use for pemphigus. However, it is also used off-label for immunobullous disorders and has potential in the treatment of melanoma, primary cutaneous B-cell lymphomas, atopic dermatitis, and connective tissue disorders [42].

Upper respiratory infections are one of the most common adverse effects of rituximab [43–46]. However, data regarding COVID-19 infection in rituximab users is mixed. In a Swiss survey study, the incidence of symptoms attributed to COVID-19 was similar between patients with inflammatory autoimmune diseases on rituximab and infliximab [47]. However, another cross-sectional survey study in Iran investigated SARS-CoV-2 infection risk in patients with multiple sclerosis (MS). They found that the use of B-cell depleting antibodies (as compared to non-cell depleting, non-cell trafficking inhibitor disease-modifying treatments [DMTs]) was associated with a 2.6-fold increase in the risk of having symptoms attributable to COVID-19 (RR: 3.55, 95% CI: 1.45, 8.68, *p*-value = 0.005) [48].

In addition to a possible increased risk of infection, patients taking rituximab who contract COVID-19 appear to suffer more severe outcomes. A large, multi-national cross-sectional study found that the use of rituximab in MS patients was associated with higher risks of hospitalization (aOR 2.76, 95% CI 1.87–4.07), ICU admission (aOR 4.32, 95% CI 2.27–8.23), and artificial ventilation (aOR 6.15, 95% CI 3.09–12.27) compared to other DMTs [49]. A California retrospective cohort study found that the risk of hospitalization due to COVID-19 was increased in MS patients on rituximab, but they did not have an increased risk of mortality [50]. Numerous other studies have demonstrated similar findings among patients with various immune-mediated diseases [51–53]. Another retrospective cohort study that collected data from the National COVID Cohort Collaborative also found an increased risk of death due to COVID-19 in patients using rituximab for rheumatological disease (HR 1.72, 95% CI 1.10–2.69) and for cancer (HR 2.57, 95% CI 1.86–3.56) [54].

Although rituximab is FDA-approved treatment for pemphigus, there are no specific guidelines published regarding the use of rituximab in dermatology during the COVID-19 pandemic. However, the American College of Rheumatology does provide guidelines for the use of rituximab, which are outlined in Table 1.

## Prednisone

Prednisone/prednisolone is a systemic corticosteroid used in dermatology for a variety of conditions, including various types of dermatitis, autoimmune bullous disease, sarcoidosis, lupus, and vasculitides. It can be used for short-term management of disease flares or longer-term for more chronic symptoms.

Few studies have assessed the impact of treatment with prednisone on the risk of COVID-19 infection. In an Iranian cross-sectional study of RA patients with COVID-19, treatment with prednisolone > 5 mg/d was an independent predictor of COVID-19 infection in multivariate analysis (odds ratio OR 2.58, 95% CI 1.57–4.25) [19].

More research has focused on the COVID-19 outcomes of patients on prednisone treatment. In a study from the Global Rheumatology Alliance registry, in patients with the rheumatologic disease taking prednisone dose ≥ 10 mg/day was associated with higher odds of hospitalization in a multivariable-adjustment analysis (OR 2.05, 95% CI 1.06 to 3.96) [55]. Multiple other studies have found similar findings in patients with systemic lupus erythematosus and autoimmune bullous diseases [56, 57]. In a retrospective study of patients with chronic liver disease, the authors observed a higher 28-day mortality rate in all patients on chronic corticosteroids compared to all patients, not on chronic corticosteroids (26.5% vs. 17.5%) [58].

Interestingly, several studies have shown prednisone works effectively as a therapy for COVID-19 infection. A randomized, open-label trial found that the mean length of hospital stay was significantly shorter in the population that received prednisolone vs. the population that received Lopinavir/Ritonavir in the modified intention to treat (5.5 vs. 6.4 days, *p* = 0.028) and per-protocol populations (4.4 vs. 5.8, *p* = 0.0007) [59].

Overall, data is limited and mixed regarding the impact of prednisone on COVID-19 incidence and outcomes. Guidelines regarding the use of prednisone during the pandemic are outlined in Table 1.

## Methotrexate

Methotrexate is a medication that has been used in dermatology since the 1960s. It inhibits enzymes responsible for nucleotide synthesis, thereby impairing DNA synthesis and cell replication. It also has anti-inflammatory effects. In dermatology, methotrexate is primarily used in the treatment of psoriasis, atopic dermatitis, blistering diseases, and connective tissue diseases.

Regarding infection risk, multiple randomized control trials found the use of methotrexate to be associated with increased risk of infection [60, 61]. However, a review of infection risk in rheumatoid arthritis patients found that methotrexate is unlikely to increase infection risk in this population [62]. In a meta-analysis, the risk of pneumonia in psoriasis patients was found to be very low at 0.8% [63]. Few studies have investigated the incidence of COVID-19 in patients on methotrexate; however, one large-scale cohort study found that patients on IL-17 inhibitors had comparable risk of COVID-19 infection compared to patients on methotrexate [23•].

In terms of COVID-19 outcomes, methotrexate does not seem to lead to worse outcomes compared to individuals not on methotrexate. In a small retrospective cohort study of 48 subjects, patients on methotrexate with alopecia areata were found to have significantly less severe COVID-19 based on symptoms of fever (*p* = 0.000) and cough and dyspnea (*p* = 0.01) compared to healthy controls. They also demonstrated reduced COVID-19 severity indicators such as pulmonary involvement (*p* = 0.001), ferritin level (*p* = 0.001), white blood cell count (*p* = 0.008), and CRP level (*p* = 0.006), compared to the control group [64]. Further, a comparative cohort study of over 53 million patients compared a group of patients on TNFis or methotrexate to patients on no immunomodulating treatment. After propensity matching, they found the likelihood of hospitalization due to COVID-19 to be similar between the two groups (risk ratio = 0.91 [95% CI 0.68–1.22], *p* = .5260) as well as the risk of death (risk ratio = 0.87 [95% CI 0.42–1.78], *p* = .6958) [65].

In general, methotrexate appears to be safe to continue during the COVID-19 pandemic. Guidelines regarding its use are outlined in Table 1.

## Cyclosporine

Cyclosporine is an immunomodulating agent that selectively targets T-cells. Cyclosporine is FDA approved for the treatment of psoriasis but is also used off-label to manage atopic dermatitis, blistering diseases, and connective tissue diseases.

Cyclosporine has been well-studied, and research suggests that at low doses used to treat dermatologic conditions, it does not significantly increase the risk of infection [66]. In a 12-month study of patients taking cyclosporine, none of them had reactivation or new onset of a viral infection [67, 68]. Interestingly, some in vitro studies have demonstrated that cyclosporine and other cyclophilin inhibitors may actually have an antiviral effect against coronaviruses, including SARS-CoV and MERS-CoV [69–71].

Findings regarding the severity of COVID-19 outcomes in patients taking cyclosporine are mixed. A Danish cohort study found that patients taking cyclosporine or tacrolimus for a variety of diseases had a significantly increased risk of hospitalization due to COVID-19 compared to patients, not on immunomodulating treatment (adjusted odds ratio 4.75 [95% CI 1.96 to 11.49]) (73). However, a retrospective cohort study using the Symphony Health dataset found no increased risk in psoriasis patients on cyclosporine versus topicals (adjusted odds ratio 0.91 [95% CI 0.62–1.35]) [72].

Interestingly, a nonrandomized pilot study investigated whether cyclosporine plus steroids improved outcomes in COVID-19 patients compared to steroids alone. In patients with moderate to severe disease, the mortality rate was 24% in the cyclosporine group versus 48.5% in the steroid group (*p* = 0.001) [73].

Findings are overall mixed, but most of the data suggests that cyclosporine is safe to continue during the COVID-19 pandemic. Official guidelines regarding its use are outlined in Table 1.

## Mycophenolate Mofetil

Mycophenolate Mofetil (MMF) is a medication that works by inhibiting B-cell and T-cell maturation. While FDA-approved for prophylaxis of organ rejection, it is used off-label in dermatology to treat psoriasis, dermatitides, connective tissue diseases, and blistering disorders [74].

MMF has been shown to increase bacterial and viral infection risk [75, 76]. In a clinical trial assessing MMF and steroids in patients with pemphigus vulgaris, patients who received combined steroids and MMF had increased rates of URTIs and nasopharyngitis, compared to those who received steroids alone [77].

Several studies have investigated the clinical severity of COVID-19 in patients on MMF. A nationwide Spanish cohort study of liver transplant patients found that baseline immunosuppression with MMF was associated with more severe disease (relative risk = 3.94; 95% CI 1.59–9.74; *p* = 0.003) [78]. Similarly, a French study of patients with inflammatory rheumatic and musculoskeletal disease assessed the impact of mycophenolic acid (MPA), of which MMF is a prodrug, on COVID-19 disease severity. They found that patients taking MPA had more severe COVID-19 disease (OR 8.02 (95% CI 3.35 to 19.20), *p* < 0.001), longer hospital stays (sHR 0.57 (95% CI 0.33 to 0.98), *p* = 0.040), and higher death rate (OR 11.58 (95% CI 4.10 to 32.69), *p* < 0.001) [79].

A review investigated several in vitro studies suggesting the anti-viral properties of MPA against MERS-CoV and SARS-CoV. However, in vivo studies have been less suggestive of this effect [80]. Overall, MMF seems to confer an increased risk of more severe COVID-19 and should be continued with close monitoring. Formal guidelines regarding its use are outlined in Table 1.

## Azathioprine

Azathioprine is a thiopurine medication that works by inhibiting purines and decreasing B and T-cell activity. It is indicated for rheumatoid arthritis and the prevention of organ transplant rejection. In dermatology, it is used to treat atopic dermatitis, intractable pruritus, blistering disorders, psoriasis, and other autoimmune diseases [81].

The infection risk with the use of azathioprine has been assessed in various studies. One clinical trial comparing the drug to methotrexate observed an infection rate of 64%, primarily upper airway infections, common colds, and mild skin infections [82]. However, a systematic review assessing off-label use of azathioprine in dermatology only noted a 3.2% rate of mild infections [83]. A French population-based study found that inflammatory bowel disease (IBD) patients on TNF-alpha inhibitors had a lower rate of viral infections compared to patients on azathioprine (HR 0.57; 95% CI, 0.38–0.87) [84]. However, a retrospective cohort study from the US Veterans Affairs System found no increased risk of COVID-19 in thiopurine users with IBD (OR 0.962; 95% CI 0.230–4.027; *p* = .9577) [85]. Thus, findings are mixed regarding SARS-CoV-2 infection risk in patients on azathioprine.

Other studies seem to demonstrate more severe COVID-19 outcomes in azathioprine users. A large international registry of IBD patients and COVID-19 found an increased risk of severe COVID-19 outcomes for patients on thiopurine monotherapy (adjusted OR (aOR) 4.08, 95% CI 1.73–9.61) and patients on combination therapy with TNF antagonist and thiopurine (aOR 4.01, 95% CI 1.65–9.78) [86].In published data from the SECURE-IBD registry, which surveils COVID-19 incidence and outcomes in IBD patients, patients on thiopurines had a hospitalization rate of 20% and a death rate of 2%. Patients on TNF-alpha inhibitor monotherapy had hospitalization and death rates of 8% and 0%, respectively [87]. While these numbers have not been statistically compared, the difference in them does suggest that azathioprine may confer increased risk of more severe outcomes in patients.

Given the mixed evidence, no formal guidelines have been published regarding azathioprine use during the COVID-19 pandemic. General guidelines for systemic medication use in dermatology are listed in Table 1.

## Conclusions

The COVID-19 pandemic has raised concerns about the safety of immunomodulating drugs for patients with dermatologic conditions. Evidence suggests that some immunomodulating drugs are safe to continue during the pandemic, while others, such as rituximab, JAK inhibitors, mycophenolate mofetil, and azathioprine may predispose patients to increased risk of infection and/or a more severe course of illness. In general, shared decision-making between patients and providers can be utilized in order to assess the benefits and risks on an individual basis. It is important to consider the risks of untreated dermatologic disease in such conversations while also being aware of the most up-to-date findings regarding COVID-19.

## Data Availability

All data supporting the findings of this study are available within the paper and its Supplementary Information. Because this is a review, all articles have been adequately cited and are available in the public domain.
